# Tópicos Emergentes em Insuficiência Cardíaca: Inibidores do Cotransportador Sódio-Glicose 2 (iSGLT2) na IC

**DOI:** 10.36660/abc.20210031

**Published:** 2021-02-19

**Authors:** Edimar Alcides Bocchi, Andréa Biolo, Lidia Zytynski Moura, José Albuquerque Figueiredo, Carlos Eduardo Lucena Montenegro, Denilson Campos de Albuquerque

**Affiliations:** 1 Clínica de Insuficiência CardíacaInstituto do CoraçãoUniversidade de São PauloSão PauloSPBrasilClínica de Insuficiência Cardíaca do Instituto do Coração (Incor) da Universidade de São Paulo, São Paulo, SP - Brasil; 2 Faculdade de MedicinaUniversidade Federal do Rio Grande do SulPorto AlegreRSBrasilFaculdade de Medicina da Universidade Federal do Rio Grande do Sul (UFRGS), Porto Alegre, RS - Brasil; 3 Hospital de Clínicas de Porto AlegrePorto AlegreRSBrasilHospital de Clínicas de Porto Alegre (HCPA), Porto Alegre, RS - Brasil; 4 Serviço de CardiologiaHospital Moinhos de VentoPorto AlegreRSBrasilServiço de Cardiologia do Hospital Moinhos de Vento, Porto Alegre, RS - Brasil; 5 Pontifícia Universidade Católica do ParanáCuritibaPRBrasilPontifícia Universidade Católica do Paraná, Curitiba, PR - Brasil; 6 Universidade Federal do MaranhãoSão LuísMABrasilUniversidade Federal do Maranhão, São Luís, MA - Brasil; 7 Pronto Socorro Cardiológico de PernambucoUPEPernambucoREBrasilPronto Socorro Cardiológico de Pernambuco (PROCAPE/UPE), Pernambuco, RE - Brasil; 8 Universidade do Estado do Rio de JaneiroRio de JaneiroRJBrasilUniversidade do Estado do Rio de Janeiro, Rio de Janeiro, RJ - Brasil; 9 Instituto D’Or de Pesquisa e EnsinoRio de JaneiroRJBrasilInstituto D’Or de Pesquisa e Ensino, Rio de Janeiro, RJ - Brasil

**Keywords:** insuficiência cardíaca, iSGLT2

## Possíveis mecanismos de ação

Os inibidores do cotransportador de sódio-glicose-2 (SGLT2) inibem a reabsorção da glicose no túbulo contorcido proximal, resultando em glicosúria e redução dos níveis glicêmicos. Entretanto, esse efeito não parece explicar os benefícios dos ISGLT2 em pacientes com insuficiência cardíaca (IC).^1,2^

Seu benefício também parece não ser diretamente relacionado ao efeito nos fatores de risco cardiovasculares clássicos [hipertensão arterial sistêmica, diabetes melito (DM), dislipidemia], uma vez que a redução de desfechos no estudo EMPA-REG não foi dependente do perfil metabólico/hemodinâmico basal dos pacientes ou de sua variação ao longo do estudo.^3^

Entre os mecanismos mais aceitos para explicar o modo de ação dos ISGLT2 na IC, estão a melhora na tensão parietal do ventrículo esquerdo secundário à diminuição da pré (efeito da natriurese e diurese osmótica) e pós-carga (melhora na função endotelial e redução da pressão arterial).^4-6^ Mecanismos metabólicos incluem a melhora no metabolismo e bioenergética do cardiomiócito (maior cetogênese e aumento da oferta de β-hidroxibutirato),^7^ inibição da bomba sódio-hidrogênio miocárdica (o que leva a maior concentração de cálcio na mitocôndria),^8^ redução da necrose e fibrose cardíacas (inibição da síntese de colágeno)^9^ e alterações na produção de citocinas e no tecido gorduroso epicárdico.^10^

Entretanto, ainda existem dúvidas sobre a real contribuição desses mecanismos.

Os benefícios são observados com e sem DM, colocando em dúvida o papel da cetogênese.

O efeito diurético dos ISGLT2 não foi observado no DAPA-HF (seja potencializando diuréticos ou reduzindo níveis de peptídeos natriuréticos).^11^ Dessa forma, o melhor conhecimento dos principais mecanismos ainda depende de estudos em modelos experimentais e de outros em andamento, como EMPEROR-preserved, EMPA-HEART e DELIVER.

**Figura 1:**
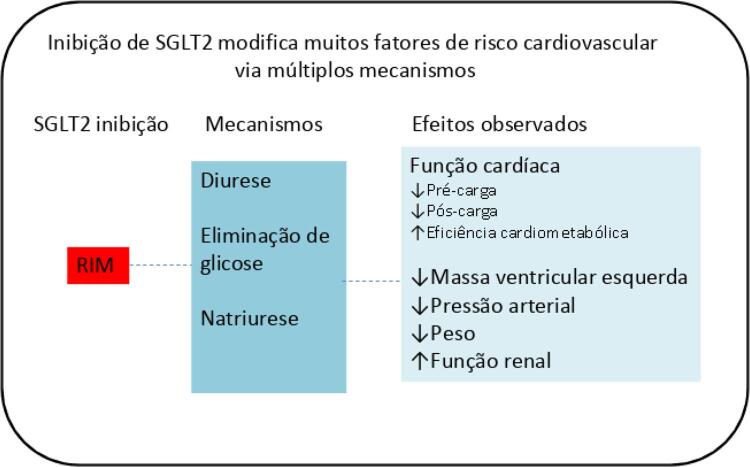
– Mecanismos de ação do iSGLT2. ISGLT2: inibidores do cotransportador de sódio-glicose-2.

Os possíveis mecanismos de ação dessa classe terapêutica estão sintetizados na Figura 1.

## Novas evidências na prevenção de IC

Em 2015, foi publicado o primeiro grande estudo dessa classe terapêutica (EMPA-REG OUTCOME),^12^ que avaliou a empagliflozina em pacientes com DM2, doença cardiovascular estabelecida e recebendo tratamento usual. Entre os pacientes que receberam empagliflozina, houve redução significativa dos principais eventos cardiovasculares adversos [MACE = morte cardiovascular (CV), infarto do miocárdio (IM) não fatal ou acidente vascular cerebral (AVC) não fatal] [*hazard ratio* (HR): 0,86; intervalo de confiança (IC) 95%: 0,74-0,99], e uma surpreendente redução de hospitalização por insuficiência cardíaca (HIC) (HR:0,65; IC 95%: 0,50-0,85). O CANVAS-Program,^13^ publicado em 2017, avaliou a canagliflozina em pacientes com DM2 com alto risco para eventos cardiovasculares e recebendo tratamento usual. Houve redução para o desfecho primário combinado (MACE = morte CV, IM não fatal ou AVC não fatal) e redução de HIC de 33% (HR= 0,67; IC 95%: 0,52-0,87) bem como dos eventos renais combinados.

O estudo DECLARE-TIMI 58^14^ avaliou a dapagliflozina em pacientes com DM2 e doença aterosclerótica estabelecida ou múltiplos fatores de risco para doença aterosclerótica e recebendo tratamento usual. Não houve redução no desfecho primário combinado (MACE = morte CV, IM ou AVC). Foi observada redução para o desfecho combinado de mortalidade cardiovascular e HIC de 17%, e uma redução de 27% (HR: 0,73; IC 95%: 0,61-0,88) para HIC. Mais recentemente o estudo Vertis–CV^15^ avaliou a ertuglifozina (ainda não comercializada no Brasil) em pacientes com DM2, doença cardiovascular estabelecida e recebendo tratamento usual. Não houve redução no desfecho primário combinado (MACE = morte CV, IM ou AVC). Foi observada, no entanto, redução de 30% na HIC.

Tomados em conjunto, os dados disponíveis demonstram a eficácia dos inibidores da SGLT2 na redução da incidência por IC em grupos de pacientes com DM2.

A avaliação de outros desfechos isoladamente evidenciou benefícios para o grupo que recebeu o medicamento. O HR foi de 0,73 (IC 95%: 0,61-0,88) para hospitalização por IC; HR de 0,83 (IC 95%: 0,73-0,95) para desfecho combinado de mortalidade CV e HIC; e HR de 0,53 (IC 95%: 0,43-0,66) para insuficiência ou mortalidade renal.

Os resultados para o desfecho primário combinado (MACE = morte CV, IM não fatal ou AVC não fatal) apontaram HR de 0,86 (IC 95%: 0,74-0,99).

## Novas evidências no tratamento da IC em pacientes com e sem DM

### Prevenção de IC no diabético

A IC é sabidamente a segunda causa de doença cardiovascular no DM2. A prevalência de IC é de 9 a 22%, o que representa quatro vezes a prevalência na população geral, sendo em geral maior no sexo feminino (redução de risco relativo (RRR) 1,95 vs 1,75).^16^ Dados recentes sugerem que o índice de massa corporal possui impacto maior no desenvolvimento de IC nos pacientes DM2 do que hemoglobina glicada por si só, diferente do desfecho IM/AVC.^16^

Assim sendo, os diferentes mecanismos das drogas hipoglicemiantes precisam ser considerados nesse desfecho de forma geral, e não apenas o controle glicêmico.^17^

Os inibidores de dipeptidil peptidase-4 (DPP-4) se mostraram neutros como classe em todos os aspectos da doença cardiovascular. Os agonistas do peptídeo semelhante a glucagon 1 (GLP1) como uma classe reduziram risco de doença cardiovascular aterosclerótica (redução de IM e/ou AVC).^18^

No entanto, foram os inibidores da SGLT2 que mostraram um benefício definitivo na redução da hospitalização por IC.^19^Recentemente, o estudo DAPA HF e EMPEROR-Reduced demonstraram em pacientes com IC com fração de ejeção reduzida ampliação dos benefícios não somente nos diabéticos, como também em não diabéticos. Nesses estudos, foram encontradas redução de internações por IC, bem como redução de mortalidade cardiovascular com uso dos iSGLT2 como terapia de adição ao tratamento farmacológico otimizado para IC.^19^

Assim, a metanálise de combinações demonstrou que um potencial regime ideal de tratamento com redução de desfechos CV e IC poderia ser a combinação de GLP1-a e SGLT2-i em um histórico de terapia com metformina.^17,19^

## iSGLT2 na ICFER - quais, para quem e em que momento

O iSGLT2 (ertugliflozin) no estudo VERTIS CV reduziu hospitalização por IC em pacientes diabéticos com doença vascular por aterosclerose.^15^ O iSGLT2 (empagliflozina) no estudo EMPEROR-Reduced reduziu o desfecho primário combinado de hospitalização por IC/morte CV; os desfechos secundários de hospitalização por IC e declínio da função renal; melhorou qualidade de vida, reduziu a hemoglobina glicada e o fragmento N-terminal do peptídeo natriurético tipo B (NT-proBNP).^20^

A dapagliflozina, no estudo DAPA-HF, reduziu o desfecho combinado de hospitalização/visita urgente por IC e morte CV; os desfechos secundários de morte cardiovascular/HIC, total de HIC/morte CV; e morte de qualquer causa e melhorou qualidade de vida.^21^ Da mesma maneira, a dapaglifozina no estudo DECLARE-TIMI reduziu evento renal^14^ e, no DAPA-CKD, reduziu o risco de sustentado declínio da função renal em pacientes com doença renal crônica, diabético ou não.^7^ Uma subanálise do estudo DAPA-HF demonstrou redução da progressão da função renal em pacientes com IC;^8^ uma análise preespecificada do EMPA-REG OUTCOME demonstrou que o ISGLT2 (empagliflozina) reduziu progressão da piora da função renal em diabéticos.^3^ No estudo CREDENCE, a canagliflozina reduziu a progressão da piora da função renal.^22^

**Figura 2:**
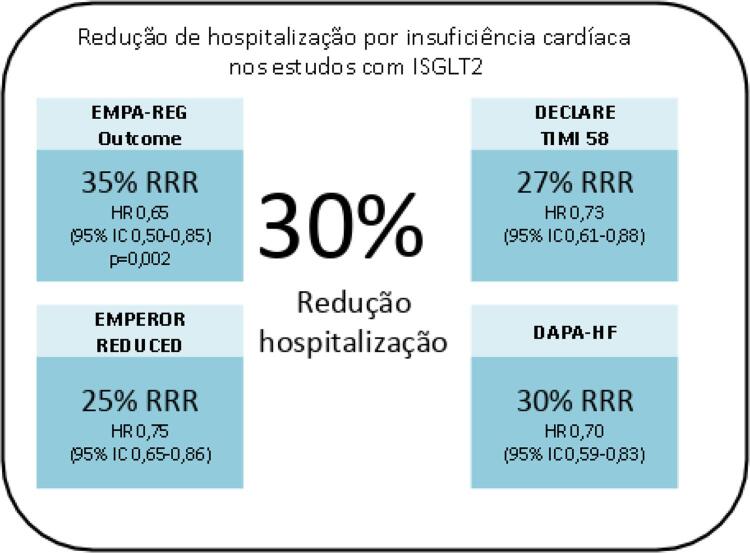
Redução de hospitalização por insuficiência cardíaca nos estudos com ISGLT2. ISGLT2: inibidores do cotransportador de sódio-glicose-2; RRR: Redução do Risco Relativo; HR: hazard ratio; IC: intervalo de confiança.

A Figura 2 sintetiza os benefícios inquestionáveis na redução de hospitalização nos principais estudos.

A última Diretriz Brasileira de Insuficiência Cardíaca da SBC, coordenada pelo Departamento de Insuficiência Cardíaca (DEIC), foi publicada em 2018 e pouco conhecimento havia sobre o papel dos iSGLT2 no manuseio terapêutico da IC.^23^ Foi consenso no DEIC que era chegado o momento de revisitá-la. Para tanto, foram realizadas reuniões preparatórias, divisões de tópicos entre os diversos colaboradores e uma reunião virtual por conta da pandemia do COVID-19 que ocorreu em 04 de dezembro de 2020. Essa reunião teve a participação de ilustres especialistas na área de IC, atualizando, opinando e incluindo novas opções terapêuticas. Os iSGLT2 foram incorporados ao manuseio terapêutico da IC reunidos em uma tabela única que está sendo publicada dentro em breve.

## Lista de Participantes do Heart Failure Summit Brazil 2020 / Departamento de Insuficiência Cardíaca - DEIC/SBC

Aguinaldo Freitas Junior, Andréia Biolo, Antonio Carlos Pereira Barretto, Antônio Lagoeiro Jorge, Bruno Biselli, Carlos Eduardo Lucena Montenegro, Denilson Campos de Albuquerque, Dirceu Rodrigues de Almeida, Edimar Alcides Bocchi, Edval Gomes dos Santos Júnior, Estêvão Lanna Figueiredo, Evandro Tinoco Mesquita, Fabiana G. Marcondes-Braga, Fábio Fernandes, Fabio Serra Silveira, Felix José Alvarez Ramires, Fernando Atik, Fernando Bacal, Flávio de Souza Brito, Germano Emilio Conceição Souza, Gustavo Calado de Aguiar Ribeiro, Humberto Villacorta Jr., Jefferson Luis Vieira, João David de Souza Neto, João Manoel Rossi Neto, José Albuquerque de Figueiredo Neto, Lídia Ana Zytynski Moura, Livia Adams Goldraich, Luís Beck-da- Silva, Luís Eduardo Paim Rohde, Luiz Claudio Danzmann, Manoel Fernandes Canesin, Marcelo Bittencourt, Marcelo Westerlund Montera, Marcely Gimenes Bonatto, Marcus Vinicius Simões, Maria da Consolação Vieira Moreira, Miguel Morita Fernandes da Silva, Monica Samuel Avila, Mucio Tavares de Oliveira Junior, Nadine Clausell, Odilson Marcos Silvestre, Otavio Rizzi Coelho Filho, Pedro Vellosa Schwartzmann, Reinaldo Bulgarelli Bestetti, Ricardo Mourilhe Rocha, Sabrina Bernadez Pereira, Salvador Rassi, Sandrigo Mangini, Silvia Marinho Martins, Silvia Moreira Ayub Ferreira, Victor Sarli Issa.
