# Supplementary material for: Tópicos Emergentes em Insuficiência Cardíaca: Inibidores do Cotransportador Sódio-Glicose 2 (iSGLT2) na IC
Source: Arq Bras Cardiol. 2021 Feb 19;116(2):355–8. [Article in Portuguese] doi: 10.36660/abc.20210031 (PMC7909964; doi:10.36660/abc.20210031)
Supplement: Translation

# Translation: Emerging Topics in Heart Failure: Sodium-Glucose Co-Transporter 2 Inhibitors (SGLT2i) in HF

**Authors:** Edimar Alcides Bocchi, Andréa Biolo, Lidia Zytynski Moura, José Albuquerque Figueiredo, Carlos Eduardo Lucena Montenegro, Denilson Campos de Albuquerque

**Affiliations:** 1 BrasilClínica de Insuficiência Cardíaca do Instituto do Coração (Incor) da Universidade de São Paulo, São Paulo, SP - Brasil; 2 BrasilFaculdade de Medicina da Universidade Federal do Rio Grande do Sul (UFRGS), Porto Alegre, RS - Brasil; 3 BrasilHospital de Clínicas de Porto Alegre (HCPA), Porto Alegre, RS - Brasil; 4 BrasilServiço de Cardiologia do Hospital Moinhos de Vento, Porto Alegre, RS - Brasil; 5 BrasilPontifícia Universidade Católica do Paraná, Curitiba, PR - Brasil; 6 BrasilUniversidade Federal do Maranhão, São Luís, MA - Brasil; 7 BrasilPronto Socorro Cardiológico de Pernambuco (PROCAPE/UPE), Pernambuco, RE - Brasil; 8 BrasilUniversidade do Estado do Rio de Janeiro, Rio de Janeiro, RJ - Brasil; 9 BrasilInstituto D’Or de Pesquisa e Ensino, Rio de Janeiro, RJ - Brasil

**Keywords:** heart failure, SGLT2i

## Possible Mechanisms of Action

Sodium-glucose co-transporter 2 inhibitors (SGLT2i) inhibit glucose reabsorption in proximal convoluted tubules, resulting in glycosuria and reduced blood glucose levels. However, this effect does not seem to explain the benefits of SGLT2i in patients with heart failure (HF).^1,2^

Its benefits also do not seem to be directly related to its effects on classic cardiovascular risk factors (SAH, DM, DLP), since outcome reduction in the EMPA-REG study was not dependent on the baseline metabolic/hemodynamic profile of the patients or their variation throughout the study.^3^

One of the most accepted mechanisms for explaining the mode of action of SGLT2i in HF is improved parietal tension of the left ventricle secondary to a decrease in pre- (effect of natriuresis and osmotic diuresis) and afterload (improvement in endothelial function and reduction of blood pressure).^4-6^ Metabolic mechanisms include improved cardiomyocyte metabolism and bioenergetics (increased ketogenesis and increased β-hydroxybutyrate levels),^7^ myocardial sodium-hydrogen pump inhibition (which leads to a higher concentrations of calcium in the mitochondria),^8^ reduced cardiac necrosis and fibrosis (inhibition of collagen synthesis)^9^ and changes in cytokine production and epicardial fatty tissue.^10^

However, there are still questions about the real contribution of these mechanisms.

Their benefits exist with or without DM, which calls the role of ketogenesis into question.

The diuretic effect of SGLT2i was not observed in DAPA-HF (either by potentiating diuretics or reducing natriuretic peptide levels).^11^ Thus, better knowledge of the main mechanisms still depends on studies in experimental models and other studies in progress, such as EMPEROR-preserved, EMPA-HEART and DELIVER.

The possible mechanisms of action of this therapeutic class are summarized in the figure below (Figure 1).

**Figure 1:** – SGLT2i: mechanisms of action.

## New evidence about HF prevention

The first large study on this therapeutic class (EMPA-REG OUTCOME) was published in 2015.^12^ It evaluated empagliflozin in DM2 patients with established cardiovascular disease who were receiving normal treatment. Among those who received empagliflozin, there was a significant reduction in major adverse cardiovascular events (MACE: CV death, non-fatal MI or non-fatal stroke) (hazard ratio [HR]: 0.86 95% CI: 0.74-0.99) and a surprising reduction in hospitalization for HF (HHF) (HR: 0.65 (95%CI: 0.50-0.85). The CANVAS Program,^13^ published in 2017, evaluated canagliflozin in DM2 patients with a high risk of cardiovascular events who were receiving normal treatment. It found a reduction in the combined primary endpoint (MACE: CV death, non-fatal MI or non-fatal stroke) and a 33% reduction in HHF (HR = 0.67, 95%CI: 0.52-0.87), as well as fewer combined renal events.

The DECLARE-TIMI 58 trial^14^ evaluated dapagliflozin in DM2 patients with established atherosclerotic disease or multiple risk factors for atherosclerotic disease who were receiving normal treatment. There was no reduction in the combined primary outcome (MACE: CV death, MI or stroke). There was a 17% reduction in the combined outcome of cardiovascular mortality and HHF, and a 27% reduction (HR: 0.73 (95%CI: 0.61-0.88) in HHF. More recently, the VERTIS–CV trial^15^ evaluated ertugliflozin (not yet marketed in Brazil) in DM2 patients with established cardiovascular disease who were receiving normal treatment. Although there was no reduction in the combined primary outcome (MACE: CV death, myocardial infarction or stroke),a 30% reduction in HHF was observed.

Taken together, the available data demonstrate the effectiveness of SGLT2i for reducing the incidence of HF in groups of DM2 patients.

When assessed in isolation, the other outcomes showed benefits for the group that received the medication. For the combined outcome of cardiovascular mortality and HHF, the HR of HHF was 0.73 (95%CI: 0.61-0.88) and the HR was 0.83 (95%CI: 0.73-0.95). For renal failure or mortality, the HR was 0.53 (95%CI: 0.43-0.66).

There was a HR of 0.86 (95%CI: 0.74-0.99)The results for the combined primary outcome (MACE: CV death, non-fatal MI or non-fatal stroke).

## New evidence about HF treatment in patients with and without DM

### HF prevention in diabetics

HF is the second leading cause of cardiovascular disease in DM2. The prevalence of HF is 9-22%, which is four times the prevalence in the general population and is generally higher in females (relative risk reduction (RRR) 1.95 vs. 1.75).^16^ Recent data suggest that, in DM2 patients, body mass index has a greater impact on the development of HF in than glycated HB itself, which is unlike the AMI/stroke outcome.^16^

Therefore, the different mechanisms of hypoglycemic drugs should be considered for this outcome in general, not just for glycemic control.^17^ It has been shown that DPP-4 inhibitors are a neutral class in all aspects of cardiovascular disease. As a class, GLP1 agonists reduced the risk of atherosclerotic cardiovascular disease (reduced AMI and/or stroke).^18^However, SGLT2i showed a definite benefit by reducing HHF.^19^ Recently, the DAPA-HF and EMPEROR-Reduced trials demonstrated increased benefits in HF patients (both diabetic and non-diabetic) with reduced ejection fraction. These studies found that, as an add-on therapy to pharmacological treatment optimized for HF, SGLT2i reduced HHF and cardiovascular mortality.^19^

Thus, a meta-analysis of combinations demonstrated that a potential ideal treatment regimen with reduced CV and HF outcomes could be a combination of GLP1-a and SGLT2i in a history of metformin therapy.^17,19^

## SGLT2i in ICFER - which, for whom, and when

In the VERTIS-CV trial, SGLT2i (ertugliflozin) reduced hospitalization for HF in diabetic patients with vascular disease due to atherosclerosis.^15^ In the EMPEROR-Reduced trial, SGLT2i (empagliflozin) reduced the combined primary outcome of HHF/cardiovascular death and the secondary outcomes HHF and decline in renal function, as well as improved quality of life, reduced glycated hemoglobin and NT-proBNP.^20^

In the DAPA-HF trial, dapagliflozin reduced the combined outcome of hospitalization/urgent visit due to HF and cardiovascular death, the secondary outcomes cardiovascular death/HHF, total HHF/cardiovascular death, all-cause mortality, and improved quality of life.^21^ Likewise, in the DECLARE-TIMI trial, dapagliflozin reduced renal events,^14^ while in the DAPA-CKD trial it reduced the risk of sustained decline in renal function in patients with chronic kidney disease, whether diabetic or not.^7^ Subanalysis of the DAPA-HF trial showed reduced progression of renal function decline in HF patients,^8^ while pre-specified analysis of the EMPA-REG OUTCOME trial showed that SGLT2i (empagliflozin) reduced the progression of renal function decline in diabetics.^3^ Finally, in the CREDENCE trial, canagliflozin reduced the progression of renal function decline.^22^

Figure 2 summarizes the unquestionable benefits of SGLT2i (i.e. reduced hospitalization) presented in the main studies.

**Figure 2:** – Reduction in hospitalization for heart failure in SGLT2i studies. RRR: relative risk reduction.

The most recent SBC Brazilian Heart Failure guideline, coordinated by the Department of Heart Failure (DHF), was published in 2018 and little was known about the role of iSGLT2 in the therapeutic management of HF.^23^ It was a consensus in the DHF that the time had come to revisit it. For that purpose, preparatory meetings were held, topic divisions were made among the different collaborators and a virtual meeting took place on December 4, 2020, due to the COVID-19 pandemic. This meeting was attended by renowned experts in the HF area, who provided updates, offered their opinions and included new therapeutic options. The iSGLT2 have been incorporated into the therapeutic management of HF, gathered together in a single table that will be published shortly.

## List of participants of the Heart Failure Summit Brazil 2020 / Heart Failure Department - Brazilian Society of Cardiology

Aguinaldo Freitas Junior, Andréia Biolo, Antonio Carlos Pereira Barretto, Antônio Lagoeiro Jorge, Bruno Biselli, Carlos Eduardo Lucena Montenegro, Denilson Campos de Albuquerque, Dirceu Rodrigues de Almeida, Edimar Alcides Bocchi, Edval Gomes dos Santos Júnior, Estêvão Lanna Figueiredo, Evandro Tinoco Mesquita, Fabiana G. Marcondes-Braga, Fábio Fernandes, Fabio Serra Silveira, Felix José Alvarez Ramires, Fernando Atik, Fernando Bacal, Flávio de Souza Brito, Germano Emilio Conceição Souza, Gustavo Calado de Aguiar Ribeiro, Humberto Villacorta Jr., Jefferson Luis Vieira, João David de Souza Neto, João Manoel Rossi Neto, José Albuquerque de Figueiredo Neto, Lídia Ana Zytynski Moura, Livia Adams Goldraich, Luís Beck-da- Silva, Luís Eduardo Paim Rohde, Luiz Claudio Danzmann, Manoel Fernandes Canesin, Marcelo Bittencourt, Marcelo Westerlund Montera, Marcely Gimenes Bonatto, Marcus Vinicius Simões, Maria da Consolação Vieira Moreira, Miguel Morita Fernandes da Silva, Monica Samuel Avila, Mucio Tavares de Oliveira Junior, Nadine Clausell, Odilson Marcos Silvestre, Otavio Rizzi Coelho Filho, Pedro Vellosa Schwartzmann, Reinaldo Bulgarelli Bestetti, Ricardo Mourilhe Rocha, Sabrina Bernadez Pereira, Salvador Rassi, Sandrigo Mangini, Silvia Marinho Martins, Silvia Moreira Ayub Ferreira, Victor Sarli Issa.
